# Effects of Motherwort Injection Versus Intramuscular Oxytocin for Preventing Postpartum Hemorrhage Among Women Who Underwent Cesarean Section

**DOI:** 10.3389/fphar.2022.859495

**Published:** 2022-03-21

**Authors:** Ming-xi Li, Chun-rong Liu, Meng Chen, Hong-cai Shang, Wen Wang, Xiao-chao Luo, Ling Li, Ya-na Qi, Yi-quan Xiong, Shi-yao Huang, Jing Wang, Kang Zou, Xing-hui Liu, Jing Tan, Xin Sun

**Affiliations:** ^1^ Chinese Evidence-based Medicine Center, West China Hospital, Sichuan University, Chengdu, China; ^2^ NMPA Key Laboratory for Real World Data Research and Evaluation in Hainan, Chengdu, China; ^3^ Sichuan Center of Technology Innovation for Real World Data, Chengdu, China; ^4^ Department of Obstetrics and Gynecology, and Key Laboratory of Birth Defects and Related Diseases of Women and Children (Sichuan University), Ministry of Education, West China Second University Hospital, Sichuan University, Chengdu, China; ^5^ Beijing University of Chinese Medicine and Pharmacology, Key Laboratory of Chinese Internal Medicine of MOE and Beijing, Beijing, China; ^6^ Sichuan Evidence-Based Medicine Center of Traditional Chinese Medicine, Hospital of Chengdu University of Traditional Chinese Medicine, Chengdu, China

**Keywords:** motherwort injection, postpartum hemorrhage, Chinese herbal medicine preparation, pregnancy registry database, real-world study

## Abstract

**Objectives:** Subject to ethical constraints, real-world data are an important resource for evaluating treatment effects of medication use during pregnancy and the postpartum period. This study investigated whether motherwort injection, a traditional Chinese medicine preparation, was more effective than intramuscular (IM) oxytocin for preventing postpartum hemorrhage (PPH) in a real-world setting when intravenous (IV) oxytocin is administered.

**Methods:** We conducted an active-controlled, propensity-score matched cohort study using an established pregnancy registry database. Women who underwent cesarean section and received IV oxytocin at the third stage of labor were included. We used an active-comparator design to minimize indication bias, in which we compared IM motherwort injection in the uterus versus IM oxytocin, both on top of IV oxytocin use. We applied 1:1 propensity-score matching (PSM) to balance patient baseline characteristics and used a logistic regression model to estimate treatment effect (i.e., risk difference (RD) and odds ratio (OR)) by using the counterfactual framework. The outcomes of interest were blood loss over 500 ml within 2 h after delivery (PPH, primary) and blood loss over 1,000 ml (severe PPH, secondary). We conducted four sensitivity analyses to examine the robustness of the results.

**Results:** A total of 22,519 pregnant women underwent cesarean sections, among which 4,081 (18.12%) PPH and 480 (2.13%) severe PPH occurred. Among included women, 586 (2.60%) were administrated with IM motherwort injection, and 21,933 (97.40%) used IM oxytocin. After PSM, patient baseline characteristics were well balanced. Compared with IM oxytocin, the use of IM motherwort injection was associated with significantly lower risk of PPH (RD −25.26%, 95% CI −30.04% to −20.47%, *p* < 0.001; OR 0.25, 95% CI 0.18 to 0.32, *p* < 0.001) and severe PPH (RD −3.58%, 95% CI −5.87% to −1.30%, *p* < 0.001; OR 0.39, 95% CI 0.20 to 0.71, *p* < 0.002). Sensitivity analyses showed that the results were similar.

**Conclusion:** With the use of data from a real-world setting, the findings consistently showed that among women undergoing cesarean section who had received IV oxytocin, the additional use of IM motherwort injection could achieve a lower risk of PPH as compared to the additional use of IM oxytocin. Our study suggested a paradigm for investigating the treatment effect of Chinese herbal medicine in the real-world practice setting.

## Introduction

Postpartum hemorrhage (PPH), defined as blood loss above 500 ml within the first 24 h after delivery, is the major cause of maternal mortality worldwide (World Health Organization, 2012). It accounts for nearly 13% of maternal deaths in developed countries and 28% in developing countries (Khan et al., 2006; World Health Organization, 2012). Uterine atony is recognized as the primary cause of PPH. Intravenous or intramuscular (IV/IM) oxytocin is recommended by World Health Organization (WHO) as the first-line treatment preventing PPH (World Health Organization, 2012). However, due to a short half-life (4–15 min), oxytocin has a short-term muscular contraction effect and may have serious adverse effects when reaching a high dose, such as tachycardia, hypotension, and water retention with hyponatremia (Pantoja et al., 2016; Pacheco et al., 2019). Thus, additional uterotonics may often be used in combination to lower the risk of PPH (Tan et al., 2016; Gallos et al., 2018).

Motherwort injection is a modern purified Chinese herbal medicine preparation derived from *Leonurus japonicus* Houtt and has a long history and a wide range of clinical use for lowering postpartum bleeding. Its main ingredients are multiple alkaloids, including leonurine and stachydrine (Ruan et al., 2003; Chen et al., 2010; Liu et al., 2017), which can stimulate contractions of uterine smooth muscle both *in vitro* and *in vivo* (Jin et al., 2004; Xie et al., 2015; Chen et al., 2019).

Motherwort injection is often IM administered to the uterus in addition to IV oxytocin (Zhao et al., 2014). In clinical practice, this Chinese herbal injection and IM oxytocin are often used in addition to IV oxytocin. These additions are added for maintaining the muscular contraction effects to lower the risk of PPH. Although earlier trials showed that the addition of motherwort injection to IV oxytocin had a better effect than IV oxytocin alone (Chen et al., 2018; Yu et al., 2019), these trials applied strict exclusion criteria, as a result of which the study population was highly selected. In addition, these trials primarily administered motherwort injection between 12 and 72 h after cesarean section, while its targeting use and effect at the point of cesarean section were not tested.

More importantly, there is no clear evidence regarding the comparative effectiveness of IM motherwort injection versus IM oxytocin to the uterus for preventing PPH when either of them is added to IV oxytocin. The lack of this evidence has a limited appropriate choice of treatment options, and guideline recommendations for this specific question are opinion based. Therefore, by using an established large pregnancy registry database, we conducted a cohort study to investigate the comparative effectiveness of IM motherwort injection versus IM oxytocin for preventing PPH during cesarean section.

## Patients and Methods

### Study Setting and Data Source

We performed a cohort study using a pregnancy registry database at West China Second University Hospital (i.e., West China Women and Children Hospital) of Sichuan University. This hospital is the leading medical center for obstetrics care and referral center in China and has an average of more than 8,000 deliveries every year (West China Second Univers). This research was conducted in accordance with the reporting of studies conducted using observational routinely collected health data statements for pharmacoepidemiology (RECORD-PE) (Langan et al., 2018).

A multidisciplinary research team composed of epidemiologists, statisticians, obstetricians, and informatics experts was organized to establish the pregnancy registry database. We included pregnant women who were registered in the first trimester and had records for any of the pregnancy outcomes, including miscarriage, induced abortion, and delivery, between January 1, 2015, and November 30, 2019. We excluded patients if they were not registered at the first trimester, lost to follow-up, or transferred to other hospitals after registration.

We used the information from electronic medical record (EMR) data to establish the database. Data extracted from the EMR system were saved separately into different files, and data linkage was performed by using a unique identifier or a combination of multiple identifiers. We used the unique patient ID number and the last menstrual period (LMP) and/or expected date of childbirth (EDC) to identify a pregnancy, thus forming a cohort from the first antenatal visit to delivery that involved data about antenatal care (e.g., pre-pregnancy conditions and prenatal visits) and hospitalization (e.g., admission examination, admission diagnosis, treatments, discharge diagnoses, etc.). We cleaned the data by using transparent and pre-specified rules, including the development of variable dictionaries, standardization of semi-structured or structured variables, identification, and handling of outliers. Finally, a preliminary multidimensional database of pregnancies registry was developed.

To validate the accuracy of the database, we reviewed all information extracted from 600 randomly selected medical records. The consistency of linkage between the pregnancy registry and hospitalization for pregnancies reached 99%. The gestational comorbidities, pregnancy complications, and maternal and fetal outcomes were recorded by the *International Classification of Diseases*, 10th edition (ICD-10). This study was approved by the Ethics Committee of West China Second University Hospital of Sichuan University (2016 No.145-03) and registered at the clinicaltrials.gov (NCT04607499).

### Study Population and Comparison

We included pregnant women who underwent cesarean section at or over 24 weeks of gestation and routinely received oxytocin by IV drip during the third stage of labor. We excluded pregnant women with incomplete information regarding the timing of cesarean section.

In order to reliably evaluate the treatment effect of motherwort injection in a real-world setting, we aimed to choose an active comparator to minimize indication bias. Thus, we compared the use of IM motherwort injection versus IM oxytocin, both of which were administered to the uterus (i.e., IM motherwort injection as the exposure group and IM oxytocin as the control group). Across all the patients, IV oxytocin was administered as a baseline treatment. Information regarding data of prescriptions and route of administration were uniformly extracted from EMR.

### Outcomes

The primary outcome of interest was the incidence of PPH, which was defined as blood loss of no less than 500 ml within the first 2 h after delivery. The amount of blood loss was uniformly measured by clinicians and nurses according to the Guideline for Management of PPH in routine clinical practice (Liu, 2014) and was recorded into the surgery records in EMR. We also investigated the severe PPH as the secondary outcome, which was diagnosed as blood loss of more than 1,000 ml within the first 2 h.

### Covariates

We selected candidate covariates based on clinical experiences and medical literature. The potentially relevant covariates were demographic and gestational characteristics, including maternal age, nation, years of education, non-permanent residents, residence of location, and pre-pregnancy body mass index (BMI), multiple gestations, parity, use of *in vitro* fertilization (IVF), and scarred uterus; gestational comorbidities, including anemia, other hematological diseases (thrombocytosis, thrombocytopenia, and antiphospholipid antibody syndrome), hepatitis virus B (HBV) infection, cardiac diseases, respiratory diseases, subclinical hypothyroidism, hyperthyroidism, pelvic diseases, and chronic hypertension during pregnancy; gestational complications, including gestational diabetes mellitus (GDM), preeclampsia, eclampsia, gestational hypertension, placenta previa, placenta implantation, intrahepatic cholestasis of pregnancy (ICP), premature rupture of membranes, polyhydramnios, oligohydramnios and fetal macrosomia; and medications during the day of cesarean section, including IV magnesium sulfate, IV dexamethasone, IM insulin, IM ademetionine, IV cephalosporins, IV lincosamides, β-lactams and β-lactamase inhibitors, IM ergometrine maleate, IV tranexamic acid, IM carboprost tromethamine injection, and IV and IV push carbetocin.

Of those, demographic characteristics were recorded in the antenatal care, and diagnosis information (i.e., comorbidities, complications, and other characteristics) was identified using the ICD-10 code, as well as surgical records, preanesthetic visits, and preoperative notes. Medications were identified from the prescription records.

### Data Analysis

We described the distribution of baseline characteristics. Given the differences in baseline characteristics and indication in medications between treatment and comparator, we used the propensity-score matching (PSM) method to achieve prognostic balances, which was conducted with a 1:1 matching ratio by using the nearest neighbor method algorithm with a caliper of 0.2 SD (Caliendo and Kopeinig, 2008; Abadie and Imbens, 2016). The propensity score (i.e., the probability of receiving IM motherwort injection) was calculated by logistic regression models of covariates mentioned above (Abadie and Imbens, 2016). We used the Matching package in the R software to estimate the risk difference (RD) and odds ratio (OR) in average treatment effect.

We evaluated the balance of baseline characteristics within the cohorts before and after propensity scoring with standardized mean difference (SMD) of characteristics distribution. If SMD between two groups was less than 0.2, a good balance between groups was achieved (Abadie and Imbens, 2016).

To validate the robustness of the results, we conducted sensitivity analyses, including the use of 1:4 matching and inverse-probability weighting (Survey package and Reshape2 package) and applying traditional multivariable logistic regression in the original full cohort. We also used blood loss above 1,000 ml in the first 24 h after delivery to verify the robustness of the outcome measure, which was recorded as a diagnosis of PPH in the summary of medical records in our hospital. All analyses were two-tailed, and a *p*-value < 0.05 was considered statistically significant. All statistical analyses were performed using R (R version R 3.4.1).

## Results

**FIGURE 1:**
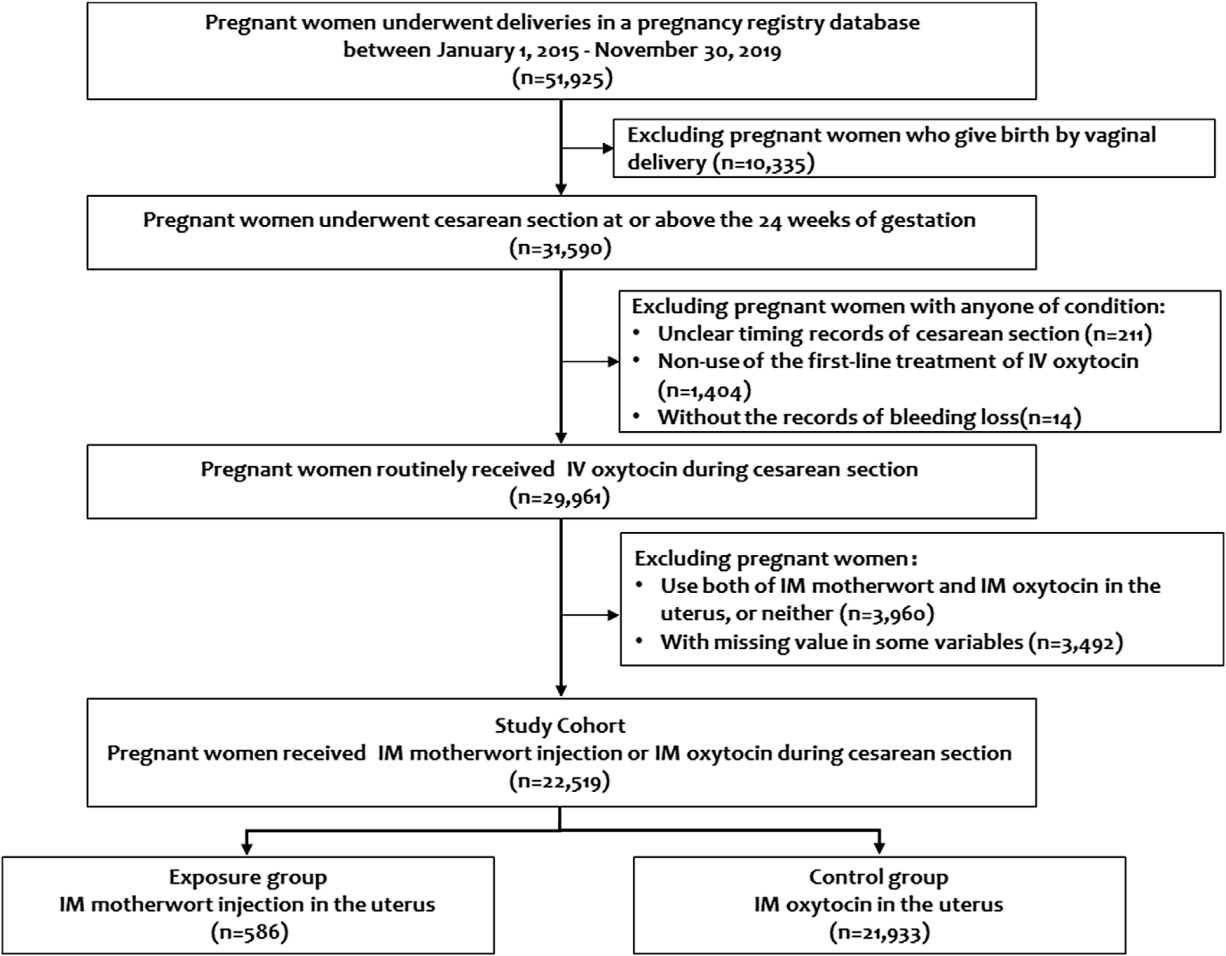
Study flowchart.

The pregnancy registry database between January 1, 2015, and December 30, 2019, included a total of 51,925 pregnant women who underwent deliveries. Of those, 29,961 pregnant women routinely received IV oxytocin during cesarean section. After exclusion of pregnant women using both IM motherwort and IM oxytocin, or neither, our cohort finally included 22,519 pregnant women, in whom 586 pregnant women received IM motherwort injection, and 21,933 received IM oxytocin. No missing values occurred among treatments and outcomes of interest (Figure 1).

### Patient Characteristics at Baseline

In the full cohort, there were significant differences in the distribution among baseline characteristics between IM motherwort injection and IM oxytocin. Before matching, pregnant women who received IM motherwort injection had a higher proportion of pelvic diseases, placenta previa, at baseline. They were also more likely to be older, to be urban residents and permanent residents, to be pregnant *via* IVF, to receive higher education, and to have more multiple pregnancies (Table 1). In addition, there were significant differences in the use of mediations during the day of cesarean section, including higher rates of IV dexamethasone, IM insulin, IV/IM cephalosporins, IM ergometrine maleate, IM carboprost tromethamine injection, and IV carbetocin among women receiving IM motherwort injection (Table 1).

**TABLE 1:** Baseline characteristics of pregnant women before and after PS matching.

	Before matching, n (%)			After matching, n (%)		
	IM motherwort injection (n = 586)	IM oxytocin (n = 21,933)	SMD	IM motherwort injection (n = 586)	IM oxytocin (n = 586)	SMD
Demographics and gestational characteristics						
Maternal age, mean (SD)	33.02 (4.26)	32.20 (4.25)	0.192	33.02 (4.26)	32.30 (3.93)	0.175
Years of education ≤12	435 (74.2)	14,876 (67.8)	0.142	435 (74.2)	428 (73.0)	0.027
Ethnic minorities	570 (97.3)	21,528 (98.2)	0.059	570 (97.3)	574 (98.0)	0.045
Urban residents	531 (90.6)	19,108 (87.1)	0.111	531 (90.6)	517 (88.2)	0.078
Non-permanent residents	54 (9.2)	3,490 (15.9)	0.203	54 (9.2)	69 (11.8)	0.084
Pre-pregnancy BMI, mean (SD)	21.12 (2.68)	21.29 (2.73)	0.065	21.12 (2.68)	21.24 (2.70)	0.045
Multiple gestations	61 (10.4)	1,471 (6.7)	0.133	61 (10.4)	90 (15.4)	0.148
Multipara	279 (47.6)	9,163 (41.8)	0.118	279 (47.6)	242 (41.3)	0.127
Use of IVF	49 (8.4)	552 (2.5)	0.260	49 (8.4)	34 (5.8)	0.100
Scarred uterus	236 (40.3)	8,112 (37.0)	0.068	236 (40.3)	195 (33.3)	0.145
Gestational complications						
Anemia	60 (10.2)	2,061 (9.4)	0.028	60 (10.2)	75 (12.8)	0.080
Other hematological diseases	14 (2.4)	1,237 (5.6)	0.166	14 (2.4)	23 (3.9)	0.088
HBV infection	31 (5.3)	1,266 (5.8)	0.021	31 (5.3)	32 (5.5)	0.008
Cardiac diseases	20 (3.4)	1,611 (7.3)	0.175	20 (3.4)	29 (4.9)	0.077
Respiratory diseases	7 (1.2)	154 (0.7)	0.051	7 (1.2)	6 (1.0)	0.016
Subclinical hypothyroidism	37 (6.3)	1,012 (4.6)	0.075	37 (6.3)	29 (4.9)	0.059
Hyperthyroidism	39 (6.7)	1,810 (8.3)	0.061	39 (6.7)	45 (7.7)	0.040
Pelvic diseases	29 (4.9)	387 (1.8)	0.177	29 (4.9)	19 (3.2)	0.086
Chronic hypertension during pregnancy	9 (1.5)	634 (2.9)	0.092	9 (1.5)	16 (2.7)	0.083
Gestational complications						
Gestational diabetes mellitus	136 (23.2)	4,619 (21.1)	0.052	136 (23.2)	112 (19.1)	0.100
Preeclampsia	16 (2.7)	614 (2.8)	0.004	16 (2.7)	24 (4.1)	0.075
Gestational hypertension	12 (2.0)	648 (3.0)	0.058	12 (2.0)	19 (3.2)	0.074
Placenta previa	76 (13.0)	1,717 (7.8)	0.169	76 (13.0)	85 (14.5)	0.045
Placenta implantation	16 (2.7)	745 (3.4)	0.039	16 (2.7)	21 (3.6)	0.049
Intrahepatic cholestasis of pregnancy	52 (8.9)	1,712 (7.8)	0.039	52 (8.9)	53 (9.0)	0.006
Premature rupture of membranes	41 (7.0)	4,077 (18.6)	0.352	41 (7.0)	74 (12.6)	0.190
Polyhydramnios	33 (5.6)	1,075 (4.9)	0.033	33 (5.6)	40 (6.8)	0.049
Oligohydramnios	13 (2.2)	831 (3.8)	0.092	13 (2.2)	10 (1.7)	0.037
Fetal macrosomia	31 (5.3)	1,738 (7.9)	0.106	31 (5.3)	39 (6.7)	0.058
Concomitant medications						
IV magnesium sulfate	26 (4.4)	1,053 (4.8)	0.017	26 (4.4)	41 (7.0)	0.110
IV dexamethasone	153 (26.1)	3,760 (17.1)	0.219	153 (26.1)	163 (27.8)	0.038
IM insulin	146 (24.9)	4,097 (18.7)	0.151	146 (24.9)	109 (18.6)	0.153
IM ademetionine	18 (3.1)	780 (3.6)	0.027	18 (3.1)	18 (3.1)	<0.001
Cephalosporins	575 (98.1)	21,027 (95.9)	0.132	575 (98.1)	569 (97.1)	0.067
Lincosamides	17 (2.9)	1,146 (5.2)	0.118	17 (2.9)	25 (4.3)	0.073
β-Lactams and β-lactamase inhibitors	9 (1.5)	217 (1.0)	0.049	9 (1.5)	10 (1.7)	0.014
IM ergometrine maleate	522 (89.1)	3,078 (14.0)	2.273	522 (89.1)	518 (88.4)	0.022
IV tranexamic acid	154 (26.3)	5,219 (23.8)	0.057	154 (26.3)	200 (34.1)	0.172
IM carboprost tromethamine injection	118 (20.1)	6,226 (28.4)	0.193	118 (20.1)	138 (23.5)	0.083
Carbetocin	575 (98.1)	3,146 (14.3)	3.152	575 (98.1)	568 (96.9)	0.077

Note. IM, intramuscular; IV, intravenous; SMD, standardized mean difference; BMI, body mass index; IVF, *in vitro* fertilization; HBV, hepatitis B virus; PS, propensity score.

After PSM, 586 (100%) pregnant women in the IM motherwort injection group were matched to those in the IM oxytocin group. In the PS-matched cohort (586:586), the differences in baseline characteristics between women who received IM motherwort injection and IM oxytocin were minimized (SMD ≤ 0.2 for all covariates) (Tables 1 and 2).

**TABLE 2:** Primary and secondary outcomes after matching.

Outcome	IM motherwort injection	IM oxytocin	Absolute risk difference (95%CI)	*p*-Value	Odds ratio (95% CI)	*p*-Value
	n (%)	n (%)				
Primary outcome						
PPH	76 (13.0)	224 (38.2)	−25.26% (−30.04%, −20.47%)	<0.001	0.25 (0.18, 0.32)	<0.001
Secondary outcome						
Severe PPH	14 (2.4)	35 (6.0)	−3.58% (−5.87%, −1.30%)	0.007	0.39 (0.20, 0.71)	0.002

Note. Propensity score (PS) matching variables: all variables in the baseline.

PPH, postpartum hemorrhage; IM, intramuscular.

### Treatment Effects by Propensity-Score Matching Analysis

The primary and secondary outcomes after matching are listed in Table 2. Among 1,172 pregnant women in the PS-matched cohort, IM motherwort injection was associated with a lower risk of blood loss during cesarean section compared with the use of IM oxytocin (for blood loss of 500 ml or more, 76/586, 13.0% versus 224/586, 38.2%; absolute RD −25.26%, 95% CI −30.04% to −20.47%; OR 0.25, 95% CI 0.18–0.32; and for blood loss ≥1,000 ml, 14/586, 2.4% versus 35/586, 6.0%; RD −3.58%, 95% CI −5.87% to −1.30%; OR 0.25, 95% CI 0.20 to 0.71).

Four sensitivity analyses showed similar results, all showing that IM motherwort injection was associated with a lower risk of blood loss during cesarean section compared with the use of IM oxytocin (Table 3).

**TABLE 3:** Sensitivity analyses.

Sensitivity analyses	Outcome	Absolute risk difference (95%CI)	*p* value	Odds ratio (95%CI)	*p* value
1:4 matching	PPH	−14.55%, 95%CI (−16.81%, −12.29%)	<0.001	0.39 (0.34, 0.46)	<0.001
	Severe PPH	−2.52%, 95%CI (−3.59%, −1.45%)	<0.001	0.47 (0.34, 0.65)	<0.001
Inverse-probability weighting	PPH	−22.19%, 95%CI (−27.72%, −16.66%)	0.005	0.29 (0.22, 0.39)	<0.001
	Severe PPH	−3.00%, 95%CI (−5.66%, −0.35%)	<0.001	0.47 (0.25, 0.86)	0.015
Traditional logistic regression	PPH	−12.94%, 95%CI (−14.29%, −11.59%)	<0.001	0.18 (0.13, 0.24)	<0.001
	Severe PPH	−1.09%, 95%CI (−1.64%, −0.54%)	0.001	0.36 (0.17, 0.69)	0.004
Blood loss ≥1,000 ml within 24 hours	—	−4.78%, 95%CI (−7.37%, −2.19%)	<0.001	0.37 (0.21, 0.64)	0.001

Note. Adjusted for all variables listed in the baseline.

PPH, postpartum hemorrhage.

## Discussion

### Interpretation of Findings and Implications

Using an established large database in the integrative medicine context, our study showed that, among women undergoing cesarean section who had received IV oxytocin as a baseline treatment, the additional use of IM motherwort injection to the uterus could achieve a lower risk of PPH compared to the additional use of IM oxytocin. Our findings were also robust to a series of sensitivity analyses, suggesting higher confidence about the findings.

Subject to ethical constraints, real-world data were generally recognized as an important data source for evaluating treatments effects for medication use during pregnancy and postpartum periods, especially for identifying the optimal treatment pattern or more benefit to the population. To the best of our knowledge, this was the first study showing that IM motherwort injection during cesarean section was more effective than IM oxytocin in the lowering of PPH risk in a real-world setting. In addition, this study provided clear evidence to clinicians for the choice of medications after IV oxytocin has been used. Our findings also supported that motherwort injection, as a traditional Chinese medicine (TCM) intervention, may have an important role in obstetric care. In particular, our study suggested that if all women had received IV oxytocin, IM motherwort injection may be a better choice than IM oxytocin, as excessive administration of oxytocin was observed to reduce oxytocin receptor availability (Gabriel et al., 2015).

Chinese herbal injections were often used in combination with western medicine as a complementary treatment. This model of practice is often complex. We have clearly demonstrated that, in the real-world setting, combination therapy was often used for preventing PPH among women undergoing cesarean section. For example, IM motherwort injection may be used on the uterus during a cesarean section on the basis of IV oxytocin. These complexities not only represent the realities of clinical practice but also require more sophisticated methods for reliably parceling out the effects of TCM interventions.

In order to achieve this purpose, we have applied an active-control design to minimize indication bias, which is usually a common and major threat to the validity of findings. We additionally applied PSM and other alternative statistical approaches in the sensitivity analyses to further control confounding. As it turned out, the consistency in findings by using different methods has strengthened the strength of inference about the effect of motherwort injection. Additionally, we particularly measured the blood loss within the first 2 h after delivery, rather than overall blood loss in earlier studies, which also has helped identify the effects of motherwort injection at the point of cesarean section.

Overall, our study not only reliably demonstrated the comparative effectiveness of motherwort injection for preventing PPH during cesarean section, but also the strategies we used in our study suggested a paradigm for investigating the treatment effect of Chinese herbal medicine in the real-world setting. The methodology used in our study represents a step forward in controlling potential confounding and indication bias, which are the two primary concerns in the assessment of treatment effects of TCM interventions.

### Comparison With Previous Studies

Previous clinical evidence including randomized controlled trials (RCTs) and their systematic reviews investigated the efficacy of motherwort injection for preventing PPH, by using different controls, such as motherwort injection plus oxytocin versus oxytocin alone, or motherwort injection versus oxytocin. Nevertheless, only a few trials compared motherwort injection versus IM oxytocin, and these trials did not find significant differences in PPH incidence. More importantly, serious methodological limitations were presented in those RCTs, such as unclear randomization and small sample sizes, thus substantially lowering the credibility of their findings (Chen et al., 2018).

In addition, our study included a broader and more heterogeneous population than previous RCTs. In comparison, previous trials excluded women if they had placenta previa, placental abruption, anemia or abnormal coagulation function, cardiopulmonary disease, previous placental adhesion, and other severe risk factors. The excluded pregnant women were typically high-risk populations; arbitrary exclusion would have hampered the generalizability of findings. Our study covered a broader population, and the findings may be applicable to clinical practice. Overall, our findings may be considered together with the existing trials to form a more inclusive body of evidence about motherwort injection.

### Mechanism of Action

Pharmacologically, motherwort injection is primarily composed of leonurine and stachydrine (Xie et al., 2015), and earlier studies have shown that its main materials include the water extraction part and *n*-butanol part (Li et al., 2014). Leonurine hydrochloride can significantly reduce the amount of vaginal bleeding and shorten bleeding time in rats by promoting frequency and tension of uterine contraction and increasing the level of serum estradiol (Xia et al., 2009). In addition, motherwort was found to cause increasing intrauterine pressure in women (Chan et al., 1983). Our findings together with earlier trials have confirmed that motherwort injection is effective for preventing PPH.

### Strengths and Limitations

Our study has a few strengths. Firstly, our study used a cohort with a large sample size and high-quality data to reliably assess the treatment effect of motherwort injection in an integrative medicine setting. To the best of our knowledge, this is the first real-world study involving more than 20,000 pregnant women to investigate the effectiveness of add-on use of motherwort injection with the first-line treatment for preventing PPH. The findings fill a gap to evaluate the effectiveness of motherwort injection in real-world practice, and the findings had reasonably good generalizability. Secondly, given the potential confounding and indication bias, we applied a novel active-controlled study design to minimize indication bias, which was common in a real-world practice setting; we also applied PS matching to construct a counterfactual framework to ensure comparability between groups, and we further conducted sensitivity analyses to consolidate the results. We believe that the use of rigorous methods and the robustness of findings through various sensitivity analyses have substantially improved the strength of findings.

Our study also has some limitations. Although we have adjusted for a variety of confounders in PSM, residual confounding from unmeasured factors may not be ruled out. Nevertheless, based on the clinical experiences and medical literature, as well as the large database, we included a large number of covariates for adjustment compared with similar studies. These factors covered a wide range of important domains that may affect the effect estimation, including demographic and gestational characteristics, gestational comorbidities, gestational complications, and concomitant medications. Residual confounding is a challenging issue in the real-world setting, and we are hopeful that other studies could validate our results. Secondly, the data of this study were derived from West China Second University Hospital, a major referral center and teaching hospital that has more than 20,000 deliveries in our cohort. The findings may not be completely generalizable to all healthcare settings.

## Conclusion

In summary, our study showed that IM motherwort injection during cesarean section was more effective than IM oxytocin in the lowering of PPH risk in a real-world setting. The findings supported that motherwort injection, as a TCM intervention, may be the better choice of medications after IV oxytocin has been used. In response to the complexities of combination therapy in an integrative medicine setting, our study suggested a paradigm for investigating the treatment effect of Chinese herbal medicine using real-world data, particularly the methodology used to control potential confounding and indication bias.

## Data Availability

The datasets presented in this article are not readily available because of privacy restrictions. The data that support the findings of the study are available from the corresponding authors upon reasonable request. Requests to access the datasets should be directed to sunxin@wchscu.cn.
